# Quantile regression of the relationship between demographic factors and leukocyte telomere length, measured by Southern blot and qPCR

**DOI:** 10.1007/s11357-025-02087-6

**Published:** 2026-01-31

**Authors:** Kyra Jennifer Waligora Mendez, Sang Kyu Lee, Casey Dagnall, Tsung-Po Lai, Hormuzd Katki, Stephen R. Spellman, Valerie Stewart, Abraham Aviv, Shahinaz M. Gadalla, Hyokyoung G. Hong

**Affiliations:** 1 Division of Cancer Epidemiology and Genetics, National Cancer Institute, Medical Center Drive, Rockville, MD 20850 USA; 2 Cancer Genomics Research Laboratory, Frederick National Laboratory for Cancer Research, Rockville, MD USA; 3 Center of Human Development and Aging, New Jersey Medical School, Rutgers, NJ USA; 4 Center for International Blood and Marrow Transplant Research, NMDP, Minneapolis, MN USA

**Keywords:** Leukocyte telomere length, Southern blotting, Quantitative polymerase chain reaction (qPCR)

## Abstract

Telomere length (TL) is associated with health outcomes. Southern Blotting (SB) is the gold standard of TL measurements, while qPCR-based TL measurements are the most used because of their high throughput. We compared leukocyte TL (LTL) measurements by SB and qPCR, and their ability to capture LTL associations with demographic factors. This study included 908 healthy donors for hematopoietic cell transplantation who had blood samples and data available at CIBMTR®. We used quantile regression (QR) to assess the associations between selected demographic factors and LTL across the LTL distribution. Pearson correlation coefficient and Bland–Altman plot were used to compare SB-LTL and qPCR-LTL measurements. SB-LTL and qPCR-LTL were modestly correlated (*r* = 0.58, *P* < 0.001). On average, SB-LTL shortened by 29 base pairs (bp) per year and was 190 bp longer in females than in males. QR analyses showed that the association between SB-LTL and age varied across the LTL distribution, with stronger age-related shortening at higher percentiles (25 bp at the 25th percentile vs. 31 bp at the 75th percentile; *P* = 0.003). Females had longer SB-LTL than males across all percentiles (*P* < 0.05 in SB-LTL analysis), and the magnitude of this sex difference did not vary significantly across the LTL distribution (157 bp at the 25th percentile vs. 221 bp at the 75th percentile; *P* = 0.33). Both SB and qPCR showed an inverse relationship between LTL and age, though the magnitudes differed between methods. Considering the full LTL distribution in studies of lifestyle factors and diseases may provide better molecular insights and guide LTL utilization in health applications.

## Introduction

Telomeres are TTAGGG DNA sequences and protein complexes located at the ends of chromosomes [1]. They shorten each time a somatic cell divides [2]. Telomere length (TL) is heritable [3–5] and is predominantly determined at birth [3]. Leukocyte TL (LTL) is shorter in older people, males, and non-Hispanic whites than African Americans [6–10]. However, inconsistencies exist between studies, likely related to differences in TL measurement methods, small sample sizes, and often both [9, 11, 12]. For example, a meta-analysis showed that quantitative polymerase chain reaction (qPCR) could not detect the significantly longer LTL in females than males, which was identified by Southern Blotting of the terminal restriction fragments (SB) [9].

SB and qPCR are common TL measurement methods [13, 14]. SB requires a large amount of DNA and generates data expressed in absolute TL units (base pairs (bp) and kilobases (kb)). In contrast, qPCR requires a small amount of DNA and estimates TL from the amplified telomere sequence to that of a single-copy gene (T/S). Thus, qPCR does not generate data in absolute TL units. While qPCR is high-throughput, relatively inexpensive, and widely used, the method is imprecise compared to SB, and its results vary across laboratories [15]. Small studies comparing the relationship between qPCR and SB TL measurements (sample sizes ranging from 11 to 190) have reported a coefficient of determination (*R*^2^) ranging from 0.2 to 0.9 [16–19]. However, the relationship between qPCR and SB may be curvilinear [20].

Studies examining the relationship between LTL and demographic factors have traditionally used mean-based linear models [5, 7, 8, 10, 11, 21, 22], such as ordinary least squares (OLS) regression. These methods are designed to quantify the average relationship between the dependent and independent variables. However, one study suggested sex differences in the relationship between LTL and age using quantile regression (QR); this relationship was detected by flow FISH LTL measurements but not by qPCR [23]. QR examines the relationships between predictors (demographic factors in this study) and the outcome variable (LTL) across different segments of the LTL distribution (in percentiles) [24]. Thus, this study (1) examines the relationship between the distribution of LTL, as measured by SB, and selected demographic characteristics in healthy adults using quantile regression; (2) compares qPCR and SB LTL measurements; and 3) evaluates the ability of the two methods to detect LTL associations with demographic factors across the spectrum of the LTL distribution.

## Methods

### Study participants and leukocyte telomere length measurements

LTL measurements were completed for 908 healthy individuals who were donors for hematopoietic cell transplantation (HCT), with frozen blood samples and sociodemographic information available at the Center for International Blood and Marrow Transplant Research (CIBMTR®). DNA was extracted with the Mag-Bind Blood and Tissue Kit (Omega Bio-Tek) on the KingFisher Flex (ThermoFisher Scientific) instrument. As described elsewhere, LTL was measured by qPCR [14] and SB [13, 16]. For SB, Hinf I and Rsa I restriction enzymes (Roche, Mannheim, Germany) were used to digest DNA. Agarose gel (0.5%) was used to resolve the terminal restriction fragments for 16 hours. The qPCR method was modified from the original monoplex qPCR method with Telo_FP and Telo_RP assay-specific primers for telomeres (T) and 36B4_FP and 36B4_RP primers for the single-copy gene (S; 36B4) [25]. LTL was expressed as a T/S ratio standardized using internal control samples. SB measurements were performed in duplicate for a subset of 75 samples and once for the rest. The Telo and 36B4 qPCR measurements were performed in triplicate for all the samples. The intraclass correlation coefficient (ICC) was 0.98 for SB and 0.82 for qPCR.

### Statistical analysis

We used the Pearson correlation and Bland–Altman plot [26] to compare SB and qPCR LTL data. QR analysis was used to investigate the heteroscedastic associations between LTL (separately for qPCR and SB) and age and sex. We also examined LTL associations with self-reported race and ethnicity, though the sample size for certain subgroups was small due to missing information.

We conducted several OLS regression and quantile regression (QR) models, and all models included the same covariates (age, sex, and race and ethnicity). Thus, calculated coefficients for each demographic factor in all models can be interpreted as independent of others. In order to maximize our sample size for the full models, individuals with missing information on race and ethnicity were combined with those reporting race other than non-Hispanic White (NHW), African American (AA), or Hispanic or Latino (HIS) in an “other/unknown” category. We present the OLS mean-based linear associations to facilitate comparisons with literature reports. Then, we used QR and calculated coefficients within each specified quantile of LTL. The interpretation of QR coefficients is the association between LTL and each demographic factor (e.g., age) within a specified quantile of LTL (25th, 50th, and 75th percentiles).

We first presented the associations between SB-LTL absolute values (in base pairs; bp) and demographic factors for biological insights. We then standardized LTL values within each method (SB or qPCR) using *z*-scores for analyses aiming to compare the SB and qPCR results. In the models with standardized LTL, coefficients represent the change in SB-LTL or qPCR-LTL in standard deviation units. Standardization affects only the scale of the coefficients and does not alter their interpretation with respect to statistical significance or direction.

## Results

### Characteristics of study participants

The mean age of study participants was 36.9 years (standard deviation (SD), 15.5; range 1.2–76.2 years), and 63% (*n* = 571) were male. Approximately half (*n* = 517; 56.9%) of the participants identified as NHW, 8.5% (*n* = 77) AA, and 7.2% (*n* = 65) as HIS. Race/ethnicity was unknown or missing in 20.3% (*n* = 184) of the participants. Mean LTL was 7.4 kb (SD 0.83; range 4.49–10.92 kb), as measured by SB, and 0.53 T/S (SD 0.11; range 0.21–0.92), as measured by qPCR (Table 1).

**Table 1:** Demographic characteristics and leukocyte telomere length distribution of study participants

	Mean (SD) or *N* (%) Total *N* = 908
**Age**	36.9 years (15.5)
**Sex**	
Male	571 (63%)
Female	337 (37%)
**Race and ethnicity**	
Non-Hispanic, White	517 (57%)
African American, Black	77 (8.5%)
Hispanic or Latino(a)	65 (7.2%)
Unknown or not reported	249 (27.4%)
**Leukocyte telomere length**	
Southern blot	7.4 kb (0.83)
Quantitative polymerase chain reaction (qPCR)	0.53 T/S (0.11)

### Southern blotting of LTL (SB-LTL) relationship with demographic factors

**Fig. 1:**
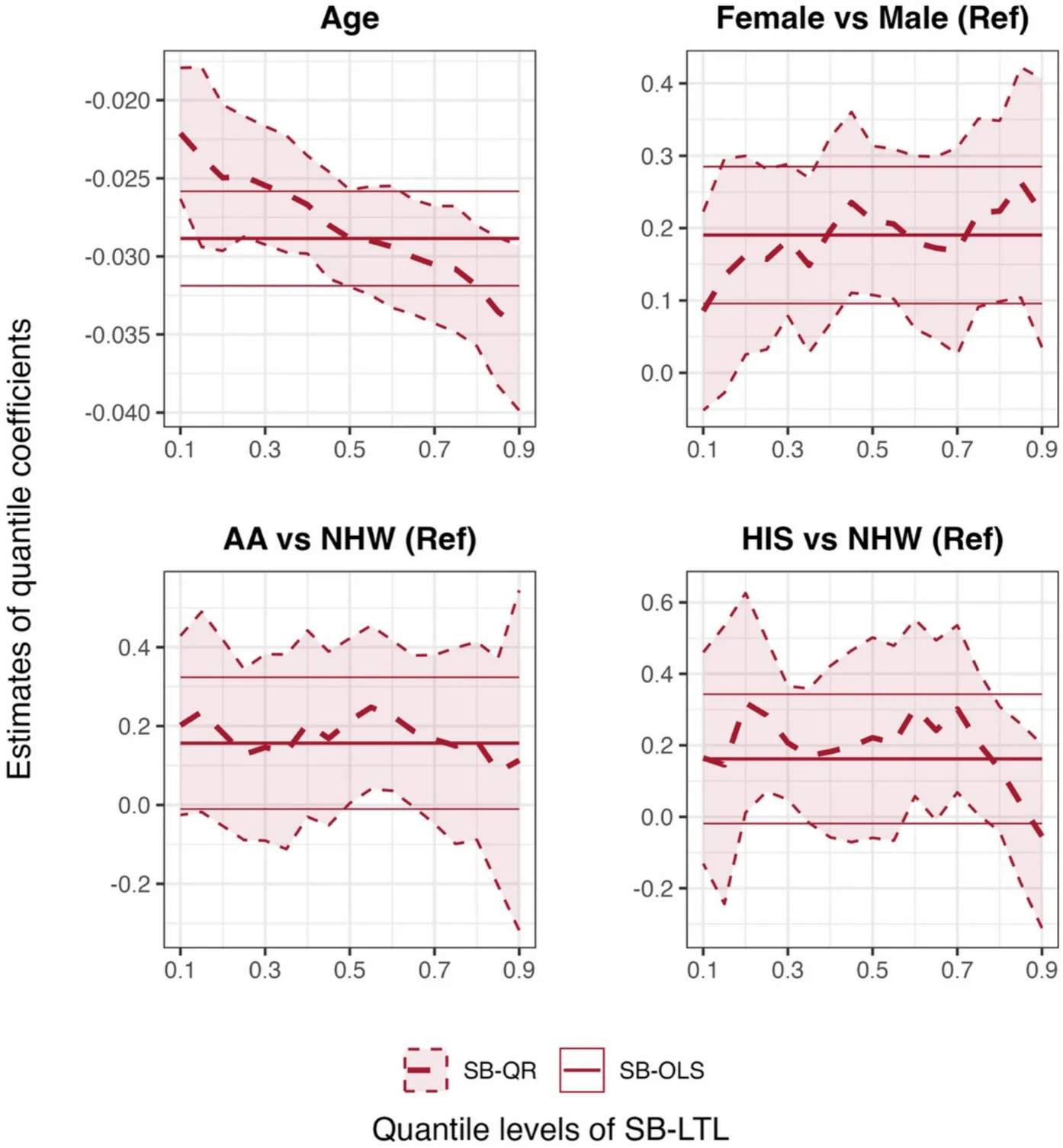
The relationship between Southern blotting of leukocyte telomere length (SB-LTL) and demographic factors. The LTL values in this model are presented in SB-LTL absolute values (unit is kilobases (kb)). The *x*-axis is LTL quantiles, and the corresponding quantile regression coefficient estimates are on the *y*-axis. The dotted lines are quantile regression (QR) coefficients with 95% confidence bands for *τ* (quantiles) 0.1 to 0.9. The solid horizontal lines are overlayed mean (OLS) regression coefficients and their 95% CIs

The OLS coefficients showed a statistically significant negative relationship between age and SB-LTL, with each additional year of age associated with a 29-bp decrease in LTL (*P* < 0.001). The quantile coefficients revealed a progressively stronger negative association between LTL and age at higher LTL quantiles (Fig. 1). The reduction in SB-LTL per year of age at the 25th percentile (shorter SB-LTL) was 25 bp, while the reduction at the 75th percentile (longer SB-LTL) was 31 bp. The difference between percentile estimated coefficients was statistically significant (*P* = 0.0034; Table 2).

**Table 2:** Ordinary least squares (OLS) and quantile regression (QR) modeling of the relationship between Southern blotting of leukocyte telomere length (SB-LTL) absolute values (units in kilobases) and demographic factors

Variable	N	OLS coefficients *β* (95% CI)	QR coefficients *β*_*τ*_ (*P*-value)		
			*τ* = 0.25	*τ* = 0.5	*τ* = 0.75
Age	908	− 0.029 (*P* < 0.001)	− 0.025 (*P* < 0.001)	− 0.029 (*P* < 0.001)	− 0.031 (*P* < 0.001)
Biological sex					
Male	571	Ref	Ref	Ref	Ref
Female	337	0.190 (*P* < 0.001)	0.157 (*P* = 0.014)	0.211 (*P* < 0.001)	0.221 (*P* < 0.001)
Race and ethnicity					
White, non-Hispanic (NHW)	517	Ref	Ref	Ref	Ref
African American or Black (AA)	77	0.157 (*P* = 0.07)	0.128 (*P* = 0.25)	0.213 (*P* = 0.04)	0.150 (*P* = 0.24)
Hispanic or Latino(a) (HIS)	65	0.162 (*P* = 0.08)	0.284 (*P* = 0.009)	0.221 (*P* = 0.12)	0.206 (*P* = 0.04)

***τ*** is the LTL quantile

The SB-LTL of females was, on average, 190 bp longer than males (*P* < 0.001). QR analysis indicated the difference between females and males was statistically significant in all SB-LTL quantiles (*P* < 0.05 for 25th, 50th, and 75th percentiles of LTL). Specifically, the difference in SB-LTL comparing females to males was 157 bp at the 25th percentile and 221 bp at the 75th percentile. However, the contrast test indicated that these two coefficients did not differ significantly from each other (*P* = 0.33), suggesting that the female–male LTL difference is constant across the LTL distribution (Fig. 1 and Table 2).

For race and ethnicity, AA or HIS individuals appeared to have longer SB-LTL than NHW individuals, although the OLS estimates were not significant, probably because of the small sample size for AA and HIS subgroups (*n* = 77 and *n* = 65, respectively). In QR, associations reached statistical significance in specific quantiles, such as SB-LTL in HIS compared to NHW at the 25th and 75th percentiles (Table 2). Yet, confidence intervals of estimated coefficients often crossed zero, suggesting that QR coefficients for race and ethnicity remained largely stable throughout the LTL distribution (Fig. 1).

### Comparing standardized z-score values of SB-LTL and qPCR-LTL and their ability to detect LTL-demographic associations

**Fig. 2:**
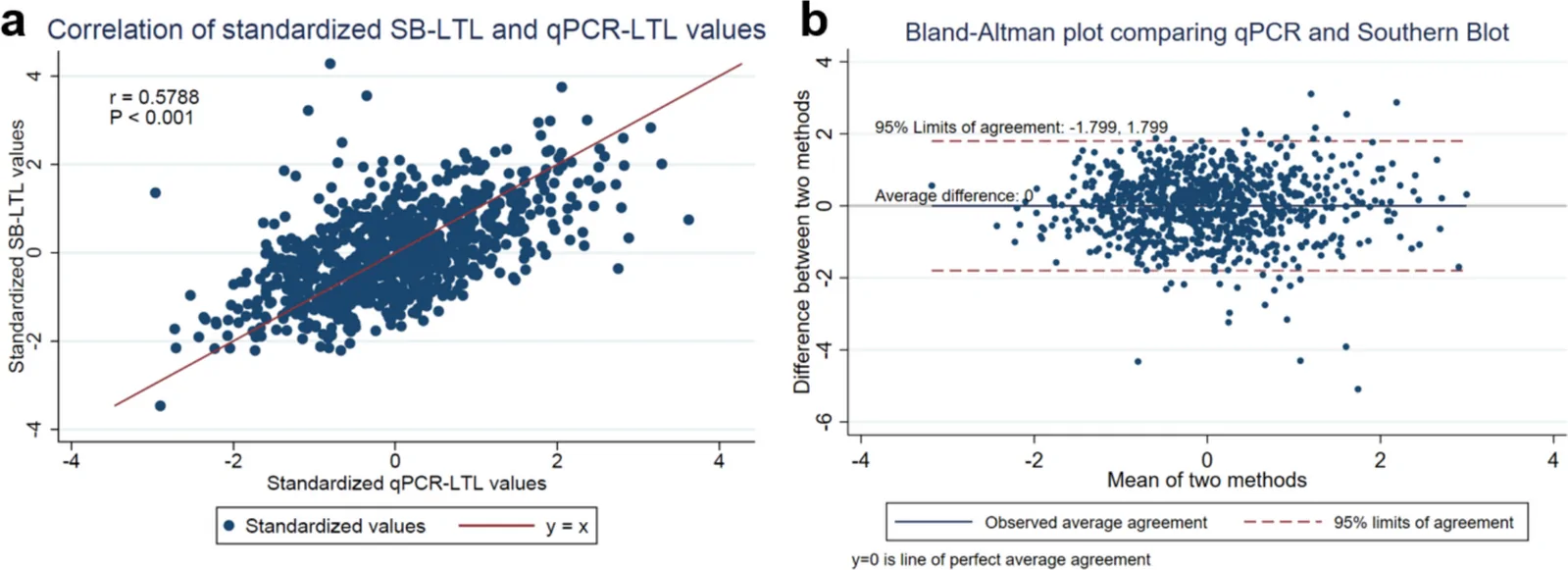
The relationship between standardized values of quantitative polymerase chain reaction leukocyte telomere length (qPCR-LTL) and Southern blotting of LTL (SB-LTL). **a)** The scatterplot of standardized qPCR-LTL and SB-LTL values with the Pearson Correlation Coefficient and **b)** the Bland–Altman Plot, where the solid line represents the average difference between the two values (qPCR and SB), and the dotted lines are the upper and lower limits of agreement

There was a moderate correlation between standardized SB-LTL and qPCR-LTL values (*r* = 0.58; 95% CI = 0.53, 0.63; *P* < 0.001; Fig. 2a). The Bland–Altman plot revealed no bias between the two methods, as shown by the random scatter of the LTL data points around the mean. Nonetheless, the limits of agreement were wide (95% limits of agreement =  − 1.799 to 1.799 SDs), indicating a considerable range within which the differences between individual measurements can fall (Fig. 2b).

Using OLS analysis, associations between LTL and selected demographics followed similar trends for both SB and qPCR measurements, with a stronger effect of age on LTL observed in SB measurements. When examining the QR coefficients, both SB-LTL and qPCR-LTL detected similar patterns between LTL and age (a negative association that increased in magnitude at higher quantiles). However, qPCR-LTL associations with age were weaker than SB-LTL in all percentiles.

**Fig. 3:**
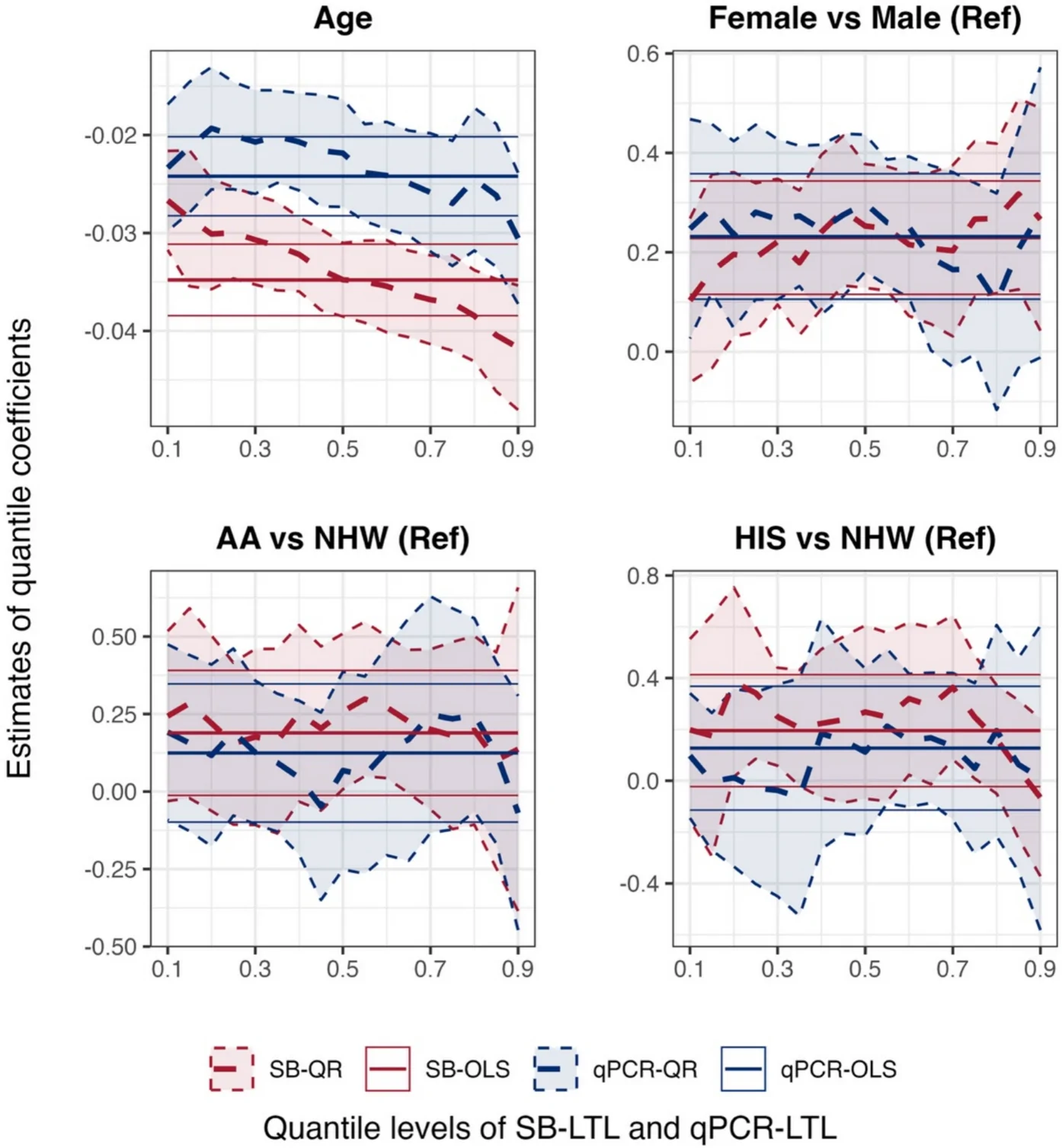
The relationship between standardized (*z*-scores) leukocyte telomere length (LTL) values and demographic factors by measurement method. Quantile regression (QR) coefficients with 95% confidence bands for 0.1 to 0.9 illustrate the heteroscedastic relationship between standardized LTL (measured by SB and qPCR) and demographic factors (age, biological sex, and race and ethnicity), including overlaid ordinary least squares (OLS) regression coefficients and their 95% CIs

Furthermore, QR results for SB-LTL and qPCR-LTL associations with sex showed expected longer LTL in females than males across percentiles but with magnitude differences in some quantiles. When examining the differences in LTL comparing AA and HIS individuals to NHW individuals, the QR coefficients for SB-LTL and qPCR-LTL differed in several quantiles (Fig. 3 and Table 3).

**Table 3:** Estimation results from ordinary least squares (OLS) and quantile regression (QR) showing the associations between standardized LTL (measured by SB and qPCR) and demographic factors (age, sex, and race and ethnicity)

Variable	LTL measurement method	OLS coefficients β (*P*-value)	QR coefficients *β*_*τ*_ (*P*-value)		
			*τ* = 0.25	*τ* = 0.5	*τ* = 0.75
Age (*n* = 908)	SB	− 0.035 (*P* < 0.001)	− 0.030 (*P* < 0.001)	− 0.035 (*P* < 0.001)	− 0.037 (*P* < 0.001)
	qPCR	− 0.024 (*P* < 0.001)	− 0.020 (*P* < 0.001)	− 0.022 (*P* < 0.001)	− 0.027 (*P* < 0.001)
Sex: Females (*n* = 337) vs*.* males (*n* = 571)	SB	0.230 (*P* < 0.001)	0.189 (*P* = 0.013)	0.254 (*P* < 0.001)	0.267 (*P* < 0.001)
	qPCR	0.232 (*P* < 0.001)	0.281 (*P* = 0.002)	0.299 (*P* < 0.001)	0.167 (*P* = 0.06)
Race and ethnicity: AA (*n* = 77) vs. NHW (*n* = 517)	SB	0.190 (*P* = 0.07)	0.154 (*P* = 0.25)	0.257 (*P* = 0.04)	0.181 (*P* = 0.24)
	qPCR	0.120 (*P* = 0.27)	0.192 (*P* = 0.16)	0.068 (*P* = 0.68)	0.234 (*P* = 0.20)
Race and ethnicity: HIS (*n* = 65) vs*.* NHW (*n* = 517)	SB	0.200 (*P* = 0.08)	0.342 (*P* = 0.009)	0.267 (*P* = 0.12)	0.248 (*P* = 0.04)
	qPCR	0.130 (*P* = 0.30)	− 0.029 (*P* = 0.88)	0.112 (*P* = 0.50)	0.048 (*P* = 0.78)

*AA* African American, *NHW* non-Hispanic White, *HIS* Hispanic or Latino

## Discussion

In this large study of healthy individuals, we replicated known LTL associations with age and sex by both SB and qPCR measurements using conventional OLS regression models. Results from quantile regression analyses revealed that the relationship between LTL and age was nonlinear and stronger in the higher LTL quantiles. These trends were observed by both SB and qPCR, although age associations with qPCR-LTL were smaller in magnitude than that of SB-LTL.

LTL and its shortening across the human lifespan have been investigated mostly using cross-sectional data. Several studies, including a recent meta-analysis, showed that there was a non-linear association between LTL and age, where LTL shortening occurs at a faster rate in younger ages and then decreases at a more stable rate in adulthood [6, 27]. Our cross-sectional data showed a 25 bp decrease in SB-LTL for each 1-year increase in age in the 25th percentile of SB-LTL *vs.* a 31 bp decrease in the 75th percentiles. This finding suggests that people who have long telomeres may experience slightly faster shortening than people with short telomeres and may be explained by a better proliferative capacity in cells with long TL. An in vitro study showed that blood cells with longer TL undergo more cell proliferation upon stimulation, while those with shorter TL had limited proliferative response [28]. In agreement with our findings, human longitudinal studies with follow-up LTL measurements at 5 years showed a positive relationship between the rate of LTL attrition and baseline LTL [29, 30]. In one study, higher LTL attrition was observed in AA people, who have longer LTL than NHW people [29]. Similarly, studies in hematopoietic cell transplantation (HCT) showed a relationship between donor LTL and the magnitude of LTL shortening 3 months after HCT [16]. On the contrary, the rate of TL shortening with age was similar in four tissues with different proliferative capacities in a cross-sectional study of adults aged 19–77 years [31]. Additional studies are warranted to understand the biological relationship between TL, proliferative needs, and attrition rate, such as testing these relationships in an experimental framework.

In agreement with previous studies, we showed that women had longer LTL than men [9]; a relationship that appeared constant across LTL quantiles, particularly in SB-LTL analysis. Studies show that the noted sex difference in LTL is largely present at birth and is proposed to be related to sex hormones, which may impact *TERT* expression, or bi-allelic expression of X chromosome genes in female embryos before implantation (reviewed in [32]). A longitudinal study showed there was no significant difference in LTL attrition between females and males, although LTL attrition in females was faster in the premenopausal period but slowed in the postmenopausal period [33]. This study lends support to the hypothesis that sex hormones may impact TL and/or attrition.

For the association between SB-LTL and race and ethnicity, our OLS models did not detect significant LTL differences among NHW, HIS, and AA individuals. The QR analyses, however, identified longer LTL in AA and HIS individuals relative to NHW individuals at specific quantiles of the LTL distribution. Prior studies have consistently reported longer LTL in AA compared with NHW using both SB and qPCR methods [7, 10]. Evidence for HIS–NHW differences is more limited, although Brown et al. reported that HIS older adults had longer LTL than NHW older adults, with a marginally significant association [7]. In our sample, African American (*n* = 77) and Hispanic or Latino (*n* = 65) individuals were much fewer than the non-Hispanic White individuals (*n* = 517), and this imbalance reduces the precision of the corresponding estimates. This limitation affects both QR and OLS, and OLS may be even more constrained because modest differences in the conditional mean are difficult to detect with limited power. QR results for the LTL relationship with race and ethnicity need to be confirmed in larger and more balanced samples.

Our findings further highlight the limitations of the qPCR-LTL measurement method, where results appeared weaker or inconsistent with SB in most findings. This is probably attributed to the wide variation between qPCR-LTL and SB-LTL measurements that was observed in our Bland–Altman analysis and that of others [34]. Previous studies have shown higher measurement error in qPCR-LTL than SB-LTL [20], with noted misclassifications particularly at the extremes (highest or lowest quartiles) or in serial samples [18, 35]. A previous study highlighted the potential effect of the relatively high measurement error when using qPCR, emphasizing a need for larger sample sizes to ensure sufficient power to detect relationships when using qPCR to measure LTL [17]. So, despite the use of *z*-score standardization to compare results from SB-LTL and qPCR-LTL, caution is needed when interpreting the findings given the expected differences in measurement reliability between the two methods.

Our study is limited by its cross-sectional nature, and validating our findings in a longitudinal study is warranted. Furthermore, we only have duplicate SB measurements for a subset of samples (*n* = 75). However, the duplicates showed excellent reliability, with an ICC of 98%. Additionally, our analyses examining LTL differences between racial and ethnic groups were exploratory and underpowered to detect modest associations. Thus, our findings require replication and validation in larger, more diverse cohorts.

In summary, our study contributes scientific insights on the relationship between qPCR and SB, as well as between LTL, age, and sex, but was limited by small sample sizes for AA and HIS individuals. The performance of the qPCR-LTL assay, particularly at the extremes of the LTL distribution, may be unreliable and should be considered when selecting methods of measurement and calculating sample sizes for new studies.

## Data Availability

Deidentified data from this study are available upon request. Data access permission will require a material transfer agreement. Requests to access the datasets should be directed to Shahinaz Gadalla, shahinaz.gadalla@nih.gov.
